# Thermodynamic Rarity Assessment of Mobile Phone PCBs: A Physical Criticality Indicator in Times of Shortage

**DOI:** 10.3390/e24010100

**Published:** 2022-01-08

**Authors:** Jorge Torrubia, Antonio Valero, Alicia Valero

**Affiliations:** Instituto CIRCE (Research Centre for Energy Resources and Consumption), Universidad de Zaragoza, 50018 Zaragoza, Spain; valero@unizar.es (A.V.); aliciavd@unizar.es (A.V.)

**Keywords:** thermodynamic rarity, resource scarcity, critical raw materials, printed circuit boards, mobile phones

## Abstract

Rising prices in energy, raw materials, and shortages of critical raw materials (CRMs) for renewable energies or electric vehicles are jeopardizing the transition to a low-carbon economy. Therefore, managing scarce resources must be a priority for governments. To that end, appropriate indicators that can identify the criticality of raw materials and products is key. Thermodynamic rarity (TR) is an exergy-based indicator that measures the scarcity of elements in the earth’s crust and the energy intensity to extract and refine them. This paper uses TR to study 70 Mobile Phone (MP) Printed Circuit Boards (PCBs) samples. Results show that an average MP PCB has a TR of 88 MJ per unit, indicating their intensive use of valuable materials. Every year the embedded TR increases by 36,250 GWh worldwide -similar to the electricity consumed by Denmark in 2019- due to annual production of MP. Pd, Ta and Au embedded in MP PCBs worldwide between 2007 and 2021 contribute to 90% of the overall TR, which account for 75, 600 and 250 tones, respectively, and increasing by 11% annually. This, coupled with the short lifespan of MP, makes PCBs an important potential source of secondary resources.

## 1. Introduction

The whole world is experiencing soaring energy and raw material costs. Europe is particularly vulnerable to this situation which must import a large part of the raw material domestically consumed by industry and households [1]. Rising energy prices -driven by fossil fuel prices [2]- (electricity [3,4,5,6], natural gas [7] and gasoline and diesel [8]), food [9,10] (fertilizers [11]) and livestock feed [12]), shipping [13,14] and even the lack of microchips for factories [15,16], are examples of this. These supply issues occur when the transformation to a low-carbon economy driven by renewables, electric vehicles, and digitization is beginning to accelerate. This situation could jeopardize the transition since a low-carbon economy requires a large quantity and variety of raw materials. For example, to produce one gigawatt (GW) of electrical power equivalent to that which a natural gas-fired power plant could supply would imply the use of approximately 160,000 tons of steel, 2000 of copper, 780 of aluminum, 110 of nickel, 85 of neodymium and 7 of dysprosium for its construction [17]. These are not negligible amounts if it is estimated that in the future, the power provided by wind turbines in 2050 could be around 2200 GW [18].

Another example is that demand for some minerals for batteries could increase dramatically by 2040 -with respect to 2020-lithium 42 times, cobalt 21 times, nickel 19 or Rare Earth Elements (REE) 7, as the International Energy Agency (IEA) warns [19]. Thus, the use of scarce minerals -needed in a low-carbon economy- could pose a problem for future generations due to their eventual depletion and unavailability in the future [20]. Furthermore, these raw materials are mainly extracted from mines that need fossil fuels to operate. The IEA’s World Energy Outlook 2021 indicates that oil and natural gas production could fall by 8–9% per year without new investments [2]. They have fallen from $779 billion in 2014 to $328 billion in 2020 [21], i.e., they have halved in 6 years. The combination of these factors could lead to bottlenecks of raw materials needed for decarbonization. Therefore, it is essential to strengthen raw material supply chains seeking alternative sources such as e-waste. According to Henckens 2021 [20], if the most stringent resource-saving measures were applied, it would be possible to extend the depletion periods of certain materials required for the energy transition by four times, even if the global service level increases.

This paper examines the raw materials embedded in printed circuit boards (PCBs) in Mobile Phones (MP) as a potential source of secondary resources. These devices have become, in recent years, irreplaceable devices for communication worldwide. The rapid growth in their sales evidences this. MP sales began to proliferate from 2009, reaching approximately constant annual sales of 1.5 billion phones between 2016 and 2020. Resulting in cumulative sales between 2007 and 2020 of almost 14 billion phones and almost doubling the world’s population [22]. The large number of MP, coupled with their short lifespan of around four years [23], contributes to the continuous increase of e-waste, which according to some projections, reach 52.2 million tons in 2021 [24] with an annual growth of 3–5%, a rate three times faster than the increase of municipal solid waste [25]. The most polluting part of a MP is the Printed Circuit Board (PCB) it contains. PCBs account for more than 70% of the carbon footprint of production [26]. In addition, it is the most heterogeneous and complicated fraction [24] as it is composed of a high diversity of elements -more than 40- and elemental concentrations [27]. Some of these elements -such as Pd, Ga or Ta- are scarce in nature [28] or a few countries control their production. Such is the case of Rare Earths Elements (REE), mainly controlled by China.

This issue has been studied by the European Commission (EC), which has been drawing up a list of Critical Raw Materials (CRMs) for the EU every three years since 2011 [29]. The EC criteria point to the economic importance for the EU economy and the supply risk of raw materials to assess criticality [28]. Such criteria are mainly based on geopolitical and economic aspects that are variable over time. For example, Si has soared 300% in less than two months [30], the volatility of Pd has been evidenced by the International Energy Agency [19], the price of Cu has increased 300% in 15 years [31] or REE prices increased 10-fold between 2009 and 2011 and then fell [32]. Moreover, the EC list has not stopped growing, the 2014 list contained 20 CRMs, the 2017 list 26 and the 2020 list 30 [33]. In addition, the list could expand in the future due to these price trends-characteristic in times of shortage-and the growing demand for metals needed for the energy transition [19].

In addition to economic and geopolitical factors, the criticality of an element is determined by its geological scarcity, as the first link in the supply chain of most CRM is mining, which will be one of the decisive factors for the success of renewable transition [19]. As mines become depleted and their ore grade decreases, the energy costs to extract the metal increase exponentially [34,35]. In the limit, a complete exhaustion of all mines would imply that the planet’s mineral wealth would be dispersed, reaching the maximum level of entropy. This state of the planet has been called Thanatia by Valero and Valero [36]. Using this reference, Thermodynamic Rarity (TR) is presented as an exergy-based indicator capable of measuring the thermodynamic criticality of raw materials based on their geological scarcity and the energy intensity required to extract, beneficiate, and refine commodities. Thus, TR is a long-term indicator decoupled from political and economic factors but constrained by mining technology and geological knowledge of the earth’s crust. More information about this indicator and its applications can be found in the following references [37,38,39,40]. TR has been proposed previously as a criticality indicator. For example, Calvo et al., 2018 [28] proposed to add Mo, Te, li, Ta, and V to the list of the 2014 EC CRMs due to their geological scarcity measured by TR. The last three have been added to the 2020 list of these elements. In addition, this indicator has already been successfully used in the study of Electrical and Electronic Equipment [41] and vehicles. Vehicle papers concluded that although Fe, Al and Cu contribute to more than 90% of the car by weight, they only account for 60% of the TR [42] and that many high TR elements end up downcycled as part of alloys or in landfills. Downcycled elements represent 4.5% of the vehicles, while in TR terms, it would be 27% [43]. Currently, EC legislation for End of Life Vehicles requires the recovery of 95% of the vehicle by weight. This can be met by recovering major metals, yet the minor ones become lost or downcycled, losing their functionality. Horta Arduin et al., 2020 [44] has also highlighted this problem in the case of display waste. They state that there is a contradiction between the EC criteria, which on the one hand is concerned with the criticality of CRMs through the publication of lists, but on the other hand, the WEEE recycling regulations focus on weight, causing many critical elements to be lost due to their low contribution in weight. This makes new indicators necessary to reinforce current regulations.

This paper is structured as follows. First, TR indicator is explained. Second, the sources used to calculate the composition of the MP PCBs, the assumptions for calculating the TR and the estimation of resources embedded worldwide are shown. Third, the results of the mass composition, TR and resources worldwide are presented. Finally, the main conclusions are discussed.

## 2. Materials and Methods

### 2.1. Thermodynamic Rarity Indicator

TR is an indicator, based on exergy, used to measure the thermodynamic criticality of raw materials, depending on their scarcity in the earth’s crust and the energy intensity associated with mining, beneficiation, and refining processes. Exergy is a property of a system relative to an associated reference state. It is the maximum work a system can deliver as it interacts with another large, but real, system, namely, a reservoir. Such a reservoir attracts the system toward degradation or entropy creation. The reference state selected for the exergy assessment of minerals is a planet, called “Thanatia” (from Greek *Thanatos*, meaning “death”) with the following characteristics [39]:

- Crust: there are no concentrated mineral deposits (the upper continental crust can be approximated to the average mineralogical composition of the current earth’s crust), fossil fuels have been depleted, and fertile soils are entirely degraded.
- Hydrosphere: its composition can be approximated to seawater since freshwater constitutes about 2.5% of global water, of which the most important part is composed of glaciers and ice sheets (68.7%) and groundwater (30.1%).
- Atmosphere: CO_2_ concentration is comparable to the complete burning of all remaining fossil fuels.

This imaginary state of the planet does not need to be “reachable”, but it is a baseline to assess the quality of any resource physically. It further allows us to objectively identify which resource is closer to depletion in the race to exhaustion. Any mineral resource with a concentration higher than that found in Thanatia has exergy, and therefore, its quality can be quantified in energy terms [39]. TR incorporates two aspects. The first is the embedded exergy cost (kJ), i.e., the useful energy required to extract and process a given mineral from the cradle to the gate (i.e., until it becomes a raw material for the manufacturing industry). The second is, in fact, an avoided cost for having minerals concentrated in mines and not dispersed throughout the crust (i.e., it can be seen as a natural bonus). As mines become depleted, it becomes exponentially harder to obtain commodities (embedded costs increase), whereas the bonus reduces. This bonus is calculated as a hypothetical exergy cost required if the given mineral would be restored to its initial composition conditions and concentration in the original mines from an utterly dispersed state, i.e., its state in Thanatia. This is the exergy replacement cost (ERC) (kJ) and can be seen as a grave-to-cradle-approach [36] or as a natural avoided exergy cost, i.e., as a natural bonus. Thus, the TR is presented as a physical indicator, stable over time, based on thermodynamic fundamentals. However, it is conditioned by mining technology, as it could reduce the exergy costs of mineral extraction and the knowledge of the earth’s crust that would modify the composition established for Thanatia. Another advantage is that it allows classifying the elements in order of criticality since each element has a unique value, measured in exergy terms.

TR values ($R_{i}$) of the analyzed elements, measured in $\frac{\mathrm{GJ}}{{\mathrm{ton}}_{i}}$ are shown in Table 1 [28]. Nevertheless, TR values could be higher than those used. As an example, Palacios et al. [45] obtained TR values 2 to 3 orders of magnitude higher than previous values for Cu and Au, using metallurgical process simulation, more specifically the HSC Chemistry software.

### 2.2. Mobile Phone PCB Data: Composition, Thermodynamic Rarity Calculation and Resources Embedded Worldwide

PCB composition has been obtained by reviewing the literature. A total of 70 samples were taken from Chancerel et al., 2009 [46] (3 samples), Kasper et al., 2011 [47] (3 samples), Oguchi et al., 2011 [48] (2 samples), Yamane et al., 2011 [49] (1 sample), Silvas et al., 2015 [50] (1 sample), Ueberschaar et al., 2017 [51] (1 sample), Ueberschaar et al., 2017 [52] (2 samples), Arshadi et al., 2018 [53] (1 sample), Holgersson et al., 2018 [54] (10 samples), Li et al., 2018 [24] (1 sample), Gu et al., 2019 [27] (12 samples), Korf et al., 2019 [55] (14 samples), Sahan et al., 2019 [25] (19 samples). All data have been transformed to mg element per kg PCB and the complete results have been compiled in Appendix A. Analyzed MP were manufactured between 2004 and 2014. PCBs were subjected to mechanical processing (shredding, comminution or milling), and then the resulting powder was analyzed using different techniques such as ICP-AES, ICP-OES, ICP-MS o XRF.

The TR of a mobile phone (MP) PCB has been calculated through Equation (1). First, the TR of a kg of PCB is calculated (in parentheses). To do this, the product of the TR of an element ($R_{i}$) by its concentration in the PCB is done and then the units of kg of PCB are transformed into units of MP.

(1) $$R_{PCB\left(unit\right)}=\left(\sum_{i=1}^{n}R_{i}\cdot \frac{mg_{i}}{kg_{PCB}}\cdot \frac{1}{1e9}\right)\;\cdot \frac{kg_{PCB}}{kg_{MP}}\cdot \frac{kg_{MP}}{Unit_{MP}}$$

Therefore, it is necessary to know its average weight and the percentage of PCBs it contains in relation to its weight. In this paper, as indicated in Equation (1), an average phone weight of 100 g and a PCB percentage by weight of 20% have been used to obtain conservative results. Table 2 shows the percentage of PCBs in phones according to different references. Equation (1) is also used to calculate the contribution of each element to the total TR to analyze the thermodynamic criticality of each element.

To estimate the mass of elements embedded in MP PCBs worldwide, the annual sales of 2020 -around 1.5 billion- and the cumulative sales between 2007 and 2021 -around 14.8 billion units- are taken [22].

Finally, the ratio between the amount of elements embedded in MP PCBs worldwide and the annual extraction of the elements is calculated. For this purpose, the quantity of each element is divided by its extraction. Thus, two percentages are obtained depending on the number of MP considered. On the one hand, the cumulative quantity is considered, i.e., 14.8 billion units between 2007 and 2021; and, on the other hand, the annual sales are considered, i.e., 1.5 billion units. Thus, the first percentage represents the annual production that could be provided if that element were recovered from all the accumulated PCBs. In addition, the second percentage of annual production could be covered with the PCBs of one year. In other words, it would be approximately the percentage of the annual production that is used to produce MP PCBs. The extraction data for the elements were obtained from the U.S. Geological Survey 2021 commodity summaries [58]. In 2020, 210 tons of Pd, 1700 tons of Ta, 3200 tons of Au, 300 tons of Ga, 20,000,000 tons of Cu, 170 tons of Pt and 900 tons of In were mined.

## 3. Results

### 3.1. Composition and Thermodynamic Rarity of Mobile Phone PCBs

**Figure 1:**
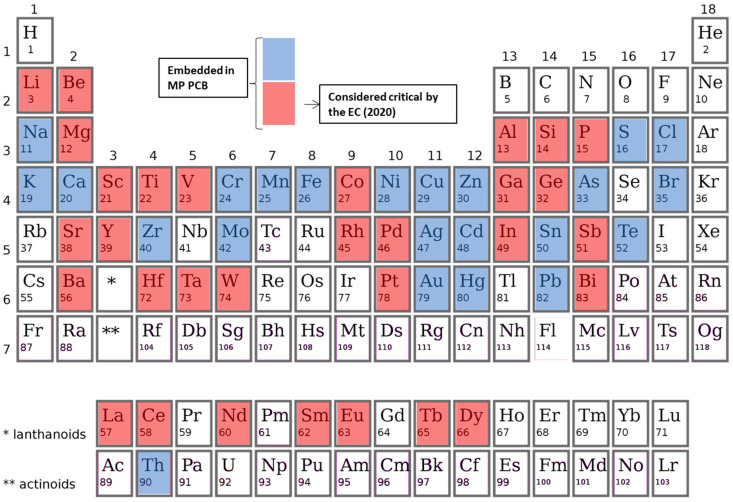
Periodic table showing the elements embedded in Mobile Phone PCBs. Elements considered critical by EC are highlighted in red.

**Figure 2:**
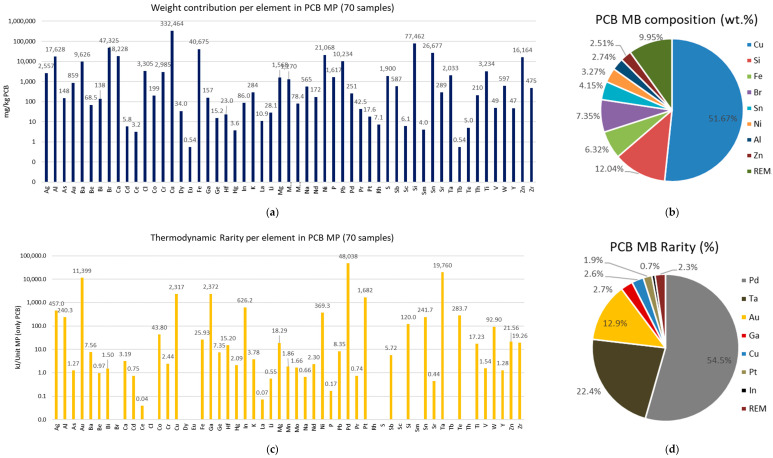
Results of composition (**a**,**b**) and thermodynamic rarity (**c**,**d**) per element.

The 70 MP PCBs samples reviewed are composed of 55 different chemical elements, of which 31 are considered as CRMs by the EC (Figure 1). Although the EC list contains 30 commodities, some of them are groups of elements such as light REE or platinum group metals (PGM), so the number obtained is greater than 30. Taking this into account, 25 elements in the MP PCB are considered critical by the EC. Nevertheless, the contribution by weight of these elements to the total PCBs is very different. Figure 2a,b shows the results of the mass contribution of each element. As can be seen, more than 90% of the weight of PCBs is made up of 8 elements: Cu, Si, Fe, Br, Sn, Ni, Al and Zn, being two CRMs according to the EC: Si and Al. Using the CE criterion, the remaining 47 elements constitute 10% of the overall weight, concentrating up to 23 CRMs. Therefore, most of the critical elements are characterized by low mass concentrations.

To measure criticality, this paper uses the TR indicator. Thus, Figure 2c shows the results of the TR contribution of each element in kJ per unit of MP and Figure 2d the results in percentage, according to the data and assumptions outlined in Section 2.2. If the TR of an element is unknown, it has been counted as 0, as for Te or Br (see Table 1). Taking TR as a criterion, the results are radically different. There are now 3 elements that contribute to almost 90% of the TR: Pd, Ta and Au, 4 others that account for 8%: Ga, Cu, Pt, and In, and remaining 48 for only 2%. Thus, seven elements account for 98% of the TR, being all of them CRMs according to the EC except Au and Cu -the most abundant in PCBs-.

**Figure 3:**
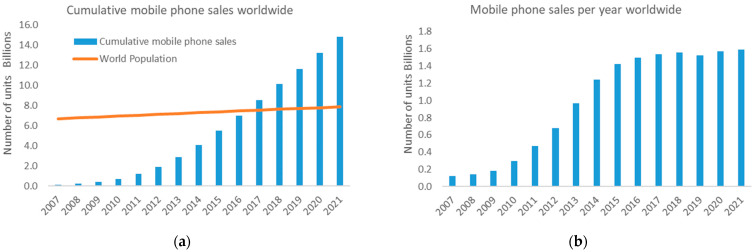
Mobile Phone sales worldwide since 2007. (**a**) Annual sales. (**b**) Cumulative sales [22].

Summing the contribution of each element as shown in Equation (1), the results indicate that the TR of a PCB is 88 MJ per MP unit. This result does not include other parts of the MP, such as the display, camera, or battery, so the TR of the complete MP is higher than obtained. Considering that between 2016 and 2020 mobile sales stagnate at around 1.5 billion mobiles per year (Figure 3b), the TR embedded in MP PCBs worldwide would increase by 1.305·10^11^ MJ o 36,250 GWh per year, an amount comparable to the electricity consumed by Denmark in 2019 [59].

### 3.2. Resources Embedded in Mobile Phone PCB Worldwide

In order to estimate the amount of resources embedded in the PCBs of MP, two sources of information have been taken. On the one hand, the number of MP sold between 2007 and 2021 is 14.8 billion, doubling the world population (Figure 3a). On the other hand, the number of MP put on sale annually considered is 1.5 billion units, which since 2016 has stagnated as shown in Figure 3b.

**Figure A1:**
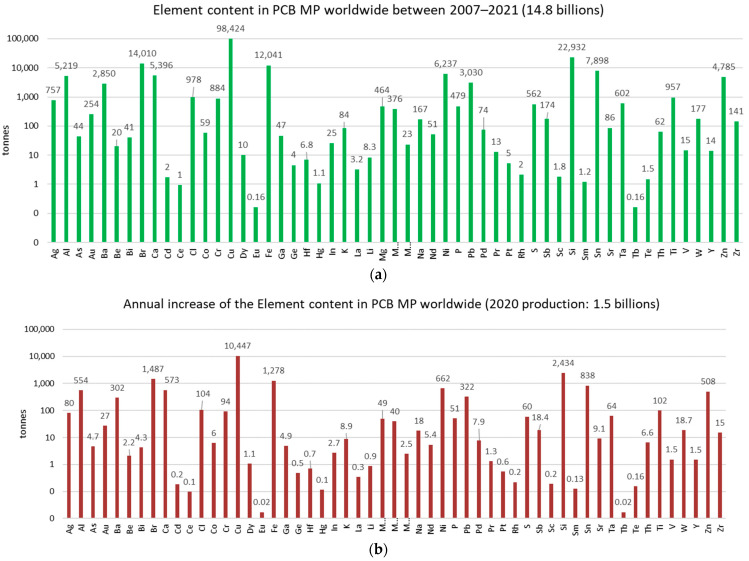
Approach of the element mass (**a**) and its annual increase (**b**) in Mobile Phone Printed Circuit Boards worldwide.

Figure A1 (Appendix A) shows the results for each element, and Table 3 shows the results for the highest TR (rows A and B). It indicates that these elements’ quantity embedded in MP PCBs increases by approximately 11% each year.

This strong annual increase and the short lifespan of the MP -of around four years [23]- make such devices an interesting source of valuable raw materials. Accordingly, we now explore how much of the annual production could -theoretically- be covered by the resources embedded in the MP PCBs. Table 3 shows the annual primary extraction of each element in row C. In the last two rows, the ratios between row A and C, and, B and C are calculated. These ratios indicate the percentage of a year’s global extraction that could be replaced if all of the embedded mass between 2007 and 2021 (A/C) could be recovered, or if all of the mass produced in one year could be recovered (B/C). It is important to emphasize that recovering the entire PCBs from MP is impossible. For example, in Reuter et al., 2018 [60], they only recover 22% of the metals from a MP in the best case. However, they achieve very high recovery rates for some elements such as Au (90–100%), Pd (10–100%) or Ga (80–90%), but much lower for others -Ta (0–10%). Another example is found in Valero-Navazo et al., 2014 [61], in which Pd, Au, Ag, Cu, Ni, Pb and Sn are recovered with recovery rates between 80 and 95%. As can be observed, the elements with higher TR are not always recovered, for example, Valero-Navazo et al., 2014 does not recover Ta or Ga, while in Reuter et al., 2018, the recovery efficiencies of Pd and Ta are 10% and Ga 80% in the worst cases. Therefore, the percentages in Table 3 should be interpreted as a theoretical maximum -unreachable- or, from another perspective, as the percentage of the extraction hoarded by the MP PCBs. In addition to the physical limitations, separate collection rates are very low, ranging from 2 to 16% [61], so high recovery rates are still far from being achieved.

Coincidentally, most elements with the highest ratios are those with the highest TR, i.e., Pd, Ta, Ga, Au, Pt and In, except for Cu. This may be due to the relationship between geological scarcity and low extraction rates. However, this should not necessarily be the case, as it is a result that depends on the composition of the devices to be analyzed. What is important to note is that the recovery of these elements should be prioritized, as they are not only the most critical from the point of view of TR, but if they were recovered, they could make an important contribution to world production. For example, in the case of Pd and Ta, their contribution to world production could theoretically reach 35% if the tons incorporated between 2007 and 2021 could be fully recovered. This figure would be 15% and 8% for Ga and Au, respectively. Considering only the tons embedded in a year, the contribution would drop to 3.8% for Pd and Ta; and to 1.7% and 0.8% for Ga and Au, respectively.

## 4. Conclusions

The volatility and increase in raw material prices and even the unavailability of some components may jeopardize the energy transition. The search for secondary raw materials and their recovery becomes necessary to alleviate this shortage situation, which could worsen in the future due to ore grade decline, among other factors. In addition, reducing primary extraction would provide other benefits such as less environmental deterioration and greater availability of resources for future generations. To this end, identifying new sources of secondary resources is essential.

This article analyzes the PCBs of the MPs, through the TR. These devices are promising candidates due to their large sales and their short useful life. The use of TR-a physical indicator based on thermodynamics allows obtaining stable values of material criticality in the medium to long term, which can only be influenced by mining technology and knowledge of the earth’s crust. This physical point of view is an essential reinforcement of the criticality assessment of any government, based on the importance of the elements for the given economy’s region and the supply risks. Being decoupled from these time-varying factors, the TR can help establish long-term policies. Another advantage of the TR is that it allows to classify and quantify the elements in order of criticality, as each element has a unique value. This helps identify products and parts with a high content of critical and valuable materials and is helpful for eco-design.

The results show that Pd, Ta, Au, Ga, Cu, Pt and In are the highest contribution to TR in MP. All are considered critical by the EC, except for Cu and Au. In addition, a considerable percentage of the world’s production of Pd, Ta, Ga and Au is hoarded in MP PCBs. These results show the need for the recovery of these elements, not only for the conservation of TR, (i.e., of the exergy embedded in the most geologically scarce elements) but also for their significant contribution to the world’s commodity production. However, 100% recovery of the resources embedded in the equipment is impossible, so to achieve the maximum recovery rate, it is necessary to develop and promote recycling processes that allow it. However, these processes are energy-intensive and require further thermodynamic analysis. This will be analyzed in a forthcoming paper.

## Figures and Tables

**Table 1:** Embedded exergy, exergy replacement cost and thermodynamic rarity of the chemical elements that compose a mobile phone PCB. * Average between ores [28].

Element (ore)	Embedded Exergy (GJ/ton)	Exergy Replacement Cost (GJ/ton)	Thermodynamic Rarity (GJ/ton)
Ag	1566	7371	8938
Al (Bauxite-Gibbsite)	54	627	682
As (Arsenopyrite)	28	400	427
Au	110,057	553,250	663,308
Ba	1	38	39
Be (Beryl)	457	253	710
Bi (Bismuthinite)	56	489	545
Cd (Greenockite)	542	5898	6440
Ce (Monazite)	523	97	620
Co (Linnaeite)	138	10,872	11,010
Cr (Chromite)	36	5	41
Cu (Chalcopyrite)	57	292	349
Fe (Hematite)	14	18	32
Ga (in Bauxite)	610,000	144,828	754,828
Gd (Monazite)	3607	478	4085
Ge (in Zinc)	498	23,750	24,248
Hf	11,183	21,814	32,997
Hg (Cinnabar)	409	28,298	28,707
In (in Zinc)	3320	360,598	363,917
K (Sylvite)	2	665	667
La (Monazite)	297	39	336
Li (Spodumene)	433	546	979
Mg (from Ocean)	10	26	36
Mn (Pyrolusite)	58	16	74
Mo (Molibdenite)	148	908	1056
Na (Halite)	43	44	87
Nd (Monazite)	592	78	670
Ni (Pentlandite and Garnierite) *	265	465	729
P (Apatite)	5	0	5
Pb (Galena)	4	37	41
Pd	583,333	8,983,377	9,566,710
Pr (Monazite)	296	577	873
Pt	291,667	4,491,690	4,783,357
Sb (Stibnite)	13	474	487
Si (Quartz)	77	1	77
Sn (Cassiterite)	27	426	453
Sr	72	4	78
Ta (Tantalite)	3091	482,828	485,919
Ti (Ilmenite and Rutile) *	196	7	203
W (Scheelite)	594	7430	8024
Y (Monazite)	1198	159	1357
Zn (Sphalerite)	42	155	197
Zr (Zircon)	1372	654	2026

**Table 2:** Percentage by weight of PCB in a Mobile Phone.

Source	[24]	[46]	[54]	[48]	[51]	[56]	[57]
Minimum	20%	22%	21%	12.6%	29.5%	21%	21.1%
Maximum	30%			30.3%			

**Table 3:** Annual increase in resources embedded in MP PCBs worldwide. Comparison between annual element production and quantity embedded in Mobile Phone PCBs. Extraction data from reference [58].

Elements		Pd	Ta	Au	Ga	Cu	Pt	In
(A) Tons embedded	[Tons]	74	602	254	46	98,423	5.2	25
(B) Tons embedded 2020	[Tons/yr]	8	64	27	5	10,500	0.6	2.7
(A)/(B) Annual increase	[%]	10.8%	10.6%	10.6%	10.9%	10.7%	11.5%	10.8%
(C) Annual primary extraction 2020	[Tons/yr]	210	1700	3200	300	20,000,000	170	900
(A)/(C)	[%]	35%	35%	7.9%	15.3%	0.49%	3.1%	2.8%
(B)/(C)	[%]	3.8%	3.8%	0.8%	1.7%	0.05%	0.4%	0.3%

## Appendix A

**Table A1:** Samples composition (from Ag to Cu) in mg of element per kg of PCB.

References (mg/kg PCB)	Ag	Al	As	Au	Ba	Be	Bi	Br	Ca	Cd	Ce	Cl	Co	Cr	Cu
Chancerel et al., 2009	2244			50											
Chancerel et al., 2009	3573			368											
Chancerel et al., 2009	5540			980											
Kasper et al., 2011	600	3100		600											395,600
Kasper et al., 2011	600	9900		1000											383,300
Kasper et al., 2011	500	6100		900											378,100
Oguchi et al., 2011	2400	67,000			4700		400						100		96,000
Oguchi et al., 2011	3800	15,000		1500	19,000		440						280		330,000
Yamane et al., 2011	2100	2600													344,900
Silvas et al., 2015	2100	2600			1600				900						355,000
Ueberschaar et al., 2017 (a)															
Ueberschaar et al., 2017 (b)	1580	19,741	0.0438	529	14,778	6	0.044		38,851	0.044	0.071		0.044		255,100
Ueberschaar et al., 2017 (b)	1597	42,979	21	1038	19,466	66	66		27,837	2	7		253		464,000
Arshadi et al., 2018	1470	57,930	480		3990			37,950	32,760			5310		1590	210,000
Holgersson et al., 2018	2640	22,736	93.3	1051	2152	98.8	39.6		1556				2.1	952.9	342,667
Holgersson et al., 2018	2773	23,003	141	1083	2662	138	60.6		762					1306.7	395,000
Holgersson et al., 2018	2500			1200						1				2000	250,000
Holgersson et al., 2018	4000			400						1				3000	200,000
Holgersson et al., 2018	4000			800						1				700	200,000
Holgersson et al., 2018	5000			1100						45				4000	120,000
Holgersson et al., 2018	5081			19						5				801	272,402
Holgersson et al., 2018	2100			10											344,900
Holgersson et al., 2018	6091			1591											590,909
Holgersson et al., 2018	3301			570											234,700
Li et al., 2018	1380	10,000		350											130,000
Gu et al., 2019	5200			600											273,700
Gu et al., 2019	600			800											385,700
Gu et al., 2019	1000			200											566,800
Gu et al., 2019	500			100											398,600
Gu et al., 2019	2300			1400											428,000
Gu et al., 2019	300			900											417,900
Gu et al., 2019	300			100											360,000
Gu et al., 2019	1100			100											408,000
Gu et al., 2019	3400														319,500
Gu et al., 2019	300			200											657,400
Gu et al., 2019															80,500
Gu et al., 2019	1300			1000											479,000
Korf et al., 2019	8118	14,949		18	10,739		5.6		33,901	5.6			64	1792	333,228
Korf et al., 2019	4125	18,333		28	6768		5.6		40,984	5.6			39	139	232,163
Korf et al., 2019	2100	2600													344,900
Korf et al., 2019	600	3100		600											395,600
Korf et al., 2019	600	9900		1000											383,300
Korf et al., 2019	500	6100		900											378,100
Korf et al., 2019	2400	67,000			4700		400						100		96,000
Korf et al., 2019	3800	15,000		1500	19,000		440						280		330,000
Korf et al., 2019	1000	32,700		600	1600			56,700	45,200			1300		200	241,900
Korf et al., 2019	430	25,200	145	1280	18,000	1.8	53.8		9480	1.7	1.7		500	123	371,000
Korf et al., 2019	310	10,600	258	1410	19,000	0.8	39		12,300	0.6	4.9		540	42,000	306,000
Korf et al., 2019	370	17,300	111	552	20,300		15.5		8340	0.9	2.1		270	650	494,000
Korf et al., 2019	2640	22,736	93.3	1051	2152	98.8	39.6		1556				2.1	952.9	342,667
Korf et al., 2019	2773	23,003	141	1083	2662	138	60.6		762					1306.7	395,000
Sahan et al., 2019	2500	8900		2400									140	190	324,700
Sahan et al., 2019	1700	13,200		650									50	110	370,400
Sahan et al., 2019	2000	10,400		2900									270	3900	227,500
Sahan et al., 2019	2000	11,500		1400									110	330	378,000
Sahan et al., 2019	4700	12,900		1300									190	510	404,000
Sahan et al., 2019	8300	10,700		1800									110	130	206,000
Sahan et al., 2019	5100	11,800		1600									120	290	287,500
Sahan et al., 2019	3700	14,900		1500									370	460	305,200
Sahan et al., 2019	3200	16,600		820									230	170	451,400
Sahan et al., 2019	3900	16,300		1100									700	440	313,100
Sahan et al., 2019	2600	20,100		530									50	440	397,700
Sahan et al., 2019	5900	15,900		1600									300	140	282,400
Sahan et al., 2019	1700	19,700		170									100	15,000	409,800
Sahan et al., 2019		6100													378,000
Sahan et al., 2019	2100	2600													344,900
Sahan et al., 2019														8500	17,700
Sahan et al., 2019	1060			65											408,000
Sahan et al., 2019	540			43											398,600
Sahan et al., 2019	4700	15,200		1400									200	400	326,200
Average	2557	17,628	148	859	9626	69	138	47,325	18,228	6	3	3305	199	2985	332,464
St deviation	1886	15,301	137	616	7663	59	178	13,258	17,311	13	3	2835	174	7838	116,836
Minimum	300	2600	0.044	10	1600	0.8	0.044	37,950	762	0.044	0.071	1300	0.044	110	17,700
Maximum	8300	67,000	480	2900	20,300	138	440	56,700	45,200	45	7	5310	700	42,000	657,400
Number of samples	66	43	10	58	18	8	15	2	14	12	5	2	27	31	66

**Table A2:** Sample composition (from Dy to Na) in mg of element per kg of PCBs.

References (mg/kg PCB)	Dy	Eu	Fe	Ga	Ge	Hf	Hg	In	K	La	Li	Mg	Mn	Mo	Na
Chancerel et al., 2009															
Chancerel et al., 2009															
Chancerel et al., 2009															
Kasper et al., 2011			14,200												
Kasper et al., 2011			65,300												
Kasper et al., 2011			48,500												
Oguchi et al., 2011			150,000												
Oguchi et al., 2011			18,000	140											
Yamane et al., 2011			105,700												
Silvas et al., 2015			124,900												
Ueberschaar et al., 2017 (a)				140											
Ueberschaar et al., 2017 (b)	0.088	0.09	13,640	0.02	0.5					0.5		0.088	0.088		
Ueberschaar et al., 2017 (b)	68	1	166,360	207	39					40		3093	997		
Arshadi et al., 2018			47,090						510			5900	5150	10	
Holgersson et al., 2018			8608				0.6		205			602	16.2	7.7	139
Holgersson et al., 2018			11,481				0.3		234			1164	23.4	8.8	753
Holgersson et al., 2018			10,000												
Holgersson et al., 2018			15,000												
Holgersson et al., 2018			20,000												
Holgersson et al., 2018			30,000												
Holgersson et al., 2018			8434				8								
Holgersson et al., 2018			26,300												
Holgersson et al., 2018			35,000												
Holgersson et al., 2018			23,500												
Li et al., 2018			50,000												
Gu et al., 2019															
Gu et al., 2019			42,700												
Gu et al., 2019			2400												
Gu et al., 2019															
Gu et al., 2019			46,000												
Gu et al., 2019															
Gu et al., 2019			10,500												
Gu et al., 2019			2800												
Gu et al., 2019			19,400												
Gu et al., 2019			15,100												
Gu et al., 2019			500												
Gu et al., 2019			5000												
Korf et al., 2019			15,089	210			12	5.6	236		13	635	654		897
Korf et al., 2019			5366	184			7	5.6	347		15	947	569		1197
Korf et al., 2019			105,700												
Korf et al., 2019			14,200												
Korf et al., 2019			65,300												
Korf et al., 2019			48,500												
Korf et al., 2019			150,000												
Korf et al., 2019			18,000	140											
Korf et al., 2019			1600						300			1900			
Korf et al., 2019			157,200	180	12.4	17.9		141		4.6	35	701	2407	244	412
Korf et al., 2019			251,000	267	20	23	0.34	134		6.3	41	1680	4900	265	400
Korf et al., 2019			18,900	103	3.9	28.2		144		2.9	37	2000	480	75	391
Korf et al., 2019			8608				0.6		205			602	16.2	7.7	139
Korf et al., 2019			11,481				0.3		234			1164	23.4	8.8	753
Sahan et al., 2019			23,600												
Sahan et al., 2019			20,400												
Sahan et al., 2019			37,200												
Sahan et al., 2019			33,900												
Sahan et al., 2019			48,400												
Sahan et al., 2019			10,000												
Sahan et al., 2019			5000												
Sahan et al., 2019			14,800												
Sahan et al., 2019			6400												
Sahan et al., 2019			11,900												
Sahan et al., 2019			46,300												
Sahan et al., 2019			10,100												
Sahan et al., 2019			34,200												
Sahan et al., 2019			48,500												
Sahan et al., 2019			105,700												
Sahan et al., 2019															
Sahan et al., 2019			2800												
Sahan et al., 2019															
Sahan et al., 2019			14,600												
Average	34	1	40,675	157	15	23	4	86	284	11	28	1568	1270	78	565
St deviation	48	1	50,004	72	15	5	5	74	104	16	13	1529	1882	111	358
Minimum	0.088	0.09	500	0.02	0.5	17.9	0.3	5.6	205	0.5	13	0.088	0.088	7.7	139
Maximum	68	1	251,000	267	39	28.2	12	144	510	40	41	5900	5150	265	1197
Number of samples	2	2	61	10	5	3	8	5	8	5	5	13	12	8	9

**Table A3:** Sample composition (from Nd to Sr) in mg of element per kg of PCBs.

References (mg/kg PCB)	Nd	Ni	P	Pb	Pd	Pr	Pt	Rh	S	Sb	Sc	Si	Sm	Sn	Sr
Chancerel et al., 2009					241										
Chancerel et al., 2009					287										
Chancerel et al., 2009					285		7								
Kasper et al., 2011		34,200		11,700										20,900	
Kasper et al., 2011		16,700		12,600										31,100	
Kasper et al., 2011		25,400		12,300										25,500	
Oguchi et al., 2011				19,000										34,000	300
Oguchi et al., 2011				13,000	300									35,000	430
Yamane et al., 2011		26,300		18,700										33,900	
Silvas et al., 2015		34,100		18,700										33,900	
Ueberschaar et al., 2017 (a)															
Ueberschaar et al., 2017 (b)	0.088	8390		3707	103	0.088	1.75			1858		108,492	0.088	17,640	
Ueberschaar et al., 2017 (b)	1162	37,628		132	56	85	1			10		112,800	8	26,948	
Arshadi et al., 2018	400	2790	1000	11,190					1900	2660		94,250		23,540	400
Holgersson et al., 2018	9.7	11,600	910	3747	119		4.3	5.7		543	0.4	66,150		19,267	108
Holgersson et al., 2018	60.7	15,433	1441	260	55.4		0.8	8.5		30.4	0.6	56,971		32,200	82.5
Holgersson et al., 2018				12,000	200					1000					
Holgersson et al., 2018				17,000	1100					200					
Holgersson et al., 2018				9000	300					500					
Holgersson et al., 2018				11,000	3					2500					
Holgersson et al., 2018				1618	5					22					
Holgersson et al., 2018				18,700											
Holgersson et al., 2018				13,636	955										
Holgersson et al., 2018				9900	294		30								
Li et al., 2018		1000		3000	210									5000	
Gu et al., 2019					400										
Gu et al., 2019		25,400		12,200										25,800	
Gu et al., 2019					100									14,000	
Gu et al., 2019		4000													
Gu et al., 2019		6000		600	100									26,000	
Gu et al., 2019		300												200	
Gu et al., 2019		8600		12,100	600										
Gu et al., 2019		3900		13,600	100									16,000	
Gu et al., 2019		27,100												17,800	
Gu et al., 2019		19,800		10,700	100										
Gu et al., 2019		100		6100										8600	
Gu et al., 2019		8000			100									20,000	
Korf et al., 2019		13,454		1405	5,6					44					334
Korf et al., 2019		6870		2495	5,6					8					372
Korf et al., 2019		26,300		18,700										33,900	
Korf et al., 2019		34,200		11,700										20,900	
Korf et al., 2019		16,700		12,600										31,100	
Korf et al., 2019		25,400		12,300										25,500	
Korf et al., 2019				19,000										34,000	300
Korf et al., 2019				13,000	300									35,000	430
Korf et al., 2019	100	2900	4000	8900					1900			104,800		14,200	500
Korf et al., 2019	44	41,500		283	99		7.3			12.2	11	60,000		38,100	284
Korf et al., 2019	32	57,000		610	178		25			9.8	12	80,200		31,400	233
Korf et al., 2019	9.5	82,900		597	100		5.3			3.16	17	45,300		41,700	372
Korf et al., 2019	9.7	11,600	910	3747	119		4.3	5.7		543	0.4	66,150		19,267	108
Korf et al., 2019	60.7	15,433	1441	260	55.4		0.8	8.5		30.4	0.6	56,971		32,200	82.5
Sahan et al., 2019		32,300		1600	10		32							62,700	
Sahan et al., 2019		13,600		1000	DL		22							34,300	
Sahan et al., 2019		23,800		7300	40		26							51,800	
Sahan et al., 2019		21,000		2600	260		28							28,300	
Sahan et al., 2019		37,700		16,700	220		50							26,200	
Sahan et al., 2019		11,000		16,300	360		33							29,600	
Sahan et al., 2019		15,800		17,900	400		19							35,500	
Sahan et al., 2019		31,900		14,600	820		7							29,700	
Sahan et al., 2019		59,300		23,300	120		12							25,300	
Sahan et al., 2019		27,000		10,000	470		15							13,000	
Sahan et al., 2019		17,000		27,300	DL		26							33,000	
Sahan et al., 2019		15,000		15,600	390		36							27,100	
Sahan et al., 2019		20,100		1900	140		26							13,700	
Sahan et al., 2019		25,400		12,300										25,500	
Sahan et al., 2019		26,300		18,700										33,900	
Sahan et al., 2019		30,200		5800											
Sahan et al., 2019		3900		13,600	50									16,000	
Sahan et al., 2019		3960													
Sahan et al., 2019		29,300		15,500	400		20							23,700	
Average	172	21,068	1617	10,234	251	43	18	7	1900	587	6	77,462	4	26,677	289
St deviation	347	16,147	1193	6859	245	60	13	2	0	895	7	23,840	6	10,939	138
Minimum	0.088	100	910	132	3	0.088	0.8	5.7	1900	3.16	0.4	45,300	0.088	200	82.5
Maximum	1162	82,900	4000	27,300	1100	85	50	8.5	1900	2660	17	112,800	8	62,700	500
Number of samples	11	52	6	59	42	2	25	4	2	17	7	11	2	50	15

**Table A4:** Sample composition (from Ta to Zr) in mg of element per kg of PCBs.

References (mg/kg PCB)	Ta	Tb	Te	Th	Ti	V	W	Y	Zn	Zr	Measured % of total PCB mass
Chancerel et al., 2009											0.25%
Chancerel et al., 2009											0.42%
Chancerel et al., 2009											0.68%
Kasper et al., 2011									34,300		51.52%
Kasper et al., 2011									9700		53.02%
Kasper et al., 2011									18,200		51.55%
Oguchi et al., 2011									8600		38.25%
Oguchi et al., 2011	2600								5000		44.45%
Yamane et al., 2011									59,200		59.34%
Silvas et al., 2015									59,200		63.30%
Ueberschaar et al., 2017 (a)											0.01%
Ueberschaar et al., 2017 (b)	897	0.088	0.044		3061				4412	0.088	49.28%
Ueberschaar et al., 2017 (b)	1006	1	10		6648				16,154	630	93.14%
Arshadi et al., 2018				210	3060				9310	370	56.12%
Holgersson et al., 2018					1640	1	111	5	5642	225	49.37%
Holgersson et al., 2018					712	0.7	122	4.1	6743	298	55.50%
Holgersson et al., 2018											27.89%
Holgersson et al., 2018											24.07%
Holgersson et al., 2018											23.53%
Holgersson et al., 2018											17.36%
Holgersson et al., 2018											28.84%
Holgersson et al., 2018											39.20%
Holgersson et al., 2018											64.82%
Holgersson et al., 2018											27.23%
Li et al., 2018											20.09%
Gu et al., 2019											27.99%
Gu et al., 2019									20,700		51.39%
Gu et al., 2019									2200		58.67%
Gu et al., 2019									4600		40.78%
Gu et al., 2019									100		51.05%
Gu et al., 2019											41.96%
Gu et al., 2019									8000		40.02%
Gu et al., 2019									4100		44.97%
Gu et al., 2019									11,900		39.91%
Gu et al., 2019									2200		70.58%
Gu et al., 2019									100		9.59%
Gu et al., 2019											51.44%
Korf et al., 2019					1508				1011		43.83%
Korf et al., 2019					708				960		32.26%
Korf et al., 2019									59,200		59.34%
Korf et al., 2019									34,300		51.52%
Korf et al., 2019									9700		53.02%
Korf et al., 2019									18,200		51.55%
Korf et al., 2019									8600		38.25%
Korf et al., 2019	2600								5000		44.45%
Korf et al., 2019					1500				700	300	52.48%
Korf et al., 2019	2800				6450	140	1110	43	3770	692	74.29%
Korf et al., 2019	2000				7100	187	860	40	5600	910	83.76%
Korf et al., 2019	2330				7300	12.8	1740	230	69,000	1280	81.70%
Korf et al., 2019					1640	1	111	5	5642	225	49.37%
Korf et al., 2019					712	0.7	122	4.1	6743	298	55.50%
Sahan et al., 2019									28,100		48.72%
Sahan et al., 2019									8200		46.36%
Sahan et al., 2019									21,200		38.83%
Sahan et al., 2019									2300		48.17%
Sahan et al., 2019									17,800		57.07%
Sahan et al., 2019									3500		29.78%
Sahan et al., 2019									7200		38.82%
Sahan et al., 2019									30,200		44.82%
Sahan et al., 2019									67,000		65.39%
Sahan et al., 2019									5100		40.30%
Sahan et al., 2019									26,900		57.19%
Sahan et al., 2019									13,600		38.81%
Sahan et al., 2019									18,900		53.54%
Sahan et al., 2019									18,200		51.40%
Sahan et al., 2019									59,200		59.34%
Sahan et al., 2019									1000		6.32%
Sahan et al., 2019									4100		44.96%
Sahan et al., 2019									4570		40.77%
Sahan et al., 2019									17,000		44.86%
Average	2033	1	5	210	3234	49	597	47	16,164	475	64.35%
St deviation	782	1	7	-	2641	79	654	82	18,616	369	
Minimum	897	0.088	0.044	210	708	0.7	111	4.1	100	0.088	
Maximum	2800	1	10	210	7300	187	1740	230	69,000	1280	
Number of samples	7	2	2	1	13	7	7	7	54	11	
